# Identification of a solitary splenic mass during surgery for gastric dilatation-volvulus is associated with diagnosis of splenic neoplasia in dogs

**DOI:** 10.3389/fvets.2026.1788740

**Published:** 2026-03-16

**Authors:** Micheala R. Polly, Steven W. Frederick, Katelyn E. Walraven

**Affiliations:** 1 Department of Surgery, BluePearl Pet Hospital, Maitland, FL, United States; 2 Medical Affairs, Mars Veterinary Health, Vancouver, WA, United States

**Keywords:** gastric dilatation and volvulus (GDV), hemangiosarcoma, histopathology, neoplasia, spectrum of care, splenectomy

## Abstract

**Objective:**

To describe associations between abnormalities in splenic tissue removed secondary to gastric dilatation-volvulus (GDV) correction and histopathologic diagnosis of splenic malignancy.

**Methods:**

Dogs treated with splenectomy secondary to GDV correction at any of 83 US veterinary referral hospitals between January 1, 2013 and May 1, 2025 were retrospectively reviewed. Data related to signalment, visual, and tactile description of the spleen during surgery, and results from the splenic histopathology report were recorded. Descriptive statistics were used to summarize histopathologic diagnoses. Frequency proportions were estimated with 95% binomial confidence intervals.

**Results:**

One hundred thirty-eight dogs were included. The median (range) weight was 31.5 (6–99.7) kg; the median (range) age was 10.1 (1.6–15.1) years. Malignancy was diagnosed in 15 of 138 (10.9%; 95% CI: 6.2%−17.3%) dogs. The prevalence of splenic malignancy did not differ significantly across age or weight quartiles. Malignancy rates significantly differed by the number of visible lesions in the spleen. There were no (0%; 95% CI: 0.0%−7.7%) malignancies in 46 dogs without visible splenic lesions, 13 (19.1%; 95% CI: 10.6%−30.5%) malignancies in 68 dogs with solitary/single visible lesions, 2 (14.3%; 95% CI: 1.8%−42.8%) malignancies in 14 dogs with two visible lesions, and 0 (0%; 95% CI: 0.0%−30.8%) malignancies in 10 dogs with 3 or more visible splenic lesions.

**Conclusion:**

The number of lesions in spleens removed secondary to GDV correction was associated with identification of splenic malignancy.

**Clinical relevance:**

The financial cost associated with splenic histopathology following splenectomy secondary to GDV correction may be warranted in spleens with one or two discrete masses.

## Introduction

Splenectomy is performed as an adjunct procedure in up to 37.4% of surgical procedures for correction of gastric dilatation-volvulus (GDV) in dogs (1). Indications for splenectomy in this context are reported to range from 8.9% to 22.6% of cases (2). Reported indications for splenectomy during GDV correction include evidence of vascular compromise or splenic pathology, such as absence of a palpable pulse in the splenic artery, tissue discoloration, or altered texture of the spleen, including discrete visible or palpable masses on or within the splenic tissue (2).

Despite the reported frequency of splenectomy procedures performed concurrent to GDV correction, no evidence has been presented to guide decision-making about processing of the removed splenic tissue in these situations. Anecdotally, clinicians may choose not to submit the removed splenic tissue for histopathologic evaluation due to a perceived low risk of malignancy in GDV cases or the added financial burden to the client.

The objective of this retrospective study was to describe the prevalence and histopathologic outcomes of splenic abnormalities in dogs undergoing GDV surgery, with the goal of informing clinicians on the likelihood of clinically significant splenic pathology and guiding decisions regarding tissue submission for histopathology in these cases. We hypothesized that the presence of at least one visible or palpable discrete mass in the splenic tissue of spleens removed secondary to GDV correction would be associated with histopathological malignancy. Further, we hypothesized that discoloration of the splenic tissue without evidence of a discrete mass would not be associated with histopathological malignancy.

## Methods

Dogs treated with splenectomy during surgical correction of GDV at any of 83 BluePearl Pet Hospitals in the US between January 1, 2013 and May 1, 2025 were retrospectively identified by searching for the invoice code for combined GDV correction and splenectomy (code: GDV + Splenectomy) using the practice management software utilized by all facilities (Cornerstone, IDEXX Laboratories, Inc, Westbrook, ME). Within the identified cohort, cases were further scrutinized to identify cases where the splenic tissue was submitted to a veterinary diagnostic laboratory (Antech Diagnostics, Fountain Valley, CA, USA) for histopathological evaluation by a veterinary pathologist.

Within that subset of dogs, all medical records related to the diagnosis, treatment, and results of the GDV and splenectomy were evaluated. Dogs were included if they had notes regarding pre-GDV health, preoperative biochemistry and CBC results, a surgical report, and a finalized splenic histopathology report. Dogs were excluded if any of the required medical record entries were missing or if the histopathology report indicated that the submitted tissue was not splenic (e.g., liver or stomach).

For all included cases, data related to signalment, pre-existing health concerns (e.g., historic surgical procedures, neoplasia), details related to the intraoperative appearance of the spleen, and any notes related to the clinician's reasoning for performing splenectomy and/or submitting the splenic tissue for histopathologic evaluation were collected. If the spleen was noted to have masses or a change of appearance, this was further categorized to dictate the number of lesions reported or described as the spleen having an atypical or abnormal color. The abnormal color was further categorized by notes about intraoperative darkening, pallor, or congestion throughout the splenic tissue.

### Statistical analysis

Descriptive statistics were used to summarize histopathologic diagnoses. Spleens with discrete splenic lesions were classified as malignant if the histopathologic diagnosis corresponded to a known malignant tumor type or benign if the histopathologic evaluation identified no evidence of malignant cells. Frequency proportions were estimated with 95% binomial confidence intervals.

Age and body weight were categorized into quartiles for analysis. Age quartiles were defined as < 8 years, 8 to < 10 years, 10 to < 12 years, and ≥12 years. Weight quarterlies were defined as < 25 kg, 25 to < 31 kg, 31 to < 40 kg, and ≥ 40 kg. Frequency distributions were compared across categories using the chi-square test of independence, except when expected frequencies were < 5 then Fisher's exact test was used. Variables potentially associated with splenic malignancy were included in a multivariable logistic regression analysis. Variable coefficients were exponentiated to produce odds ratios (OR) and corresponding 95% confidence intervals (CI). Goodness-of-fit was assessed using the Hosmer-Lemeshow test. An alpha error rate of < 0.05 was set as statistically significant.

## Results

One thousand twenty-two dogs were treated with splenectomy concurrent to GDV correction, and 187 dogs (18.3%) had tissue submitted for histopathologic evaluation. A further 49 of the 187 dogs were excluded because the tissue submitted for histopathology was not splenic, resulting in 138 dogs (13.5%) meeting all inclusion criteria. There were more males, 71 neutered and 13 intact, (60.9%; 84/138) than females, 49 spayed and 5 intact (39.1%; 54/138) in the study population. There were 37 different breeds of dogs, with the most common being mixed breed dogs (37; 26.8%), Standard Poodle (17; 12.3%), German Shepherd (14; 10.1%), Labrador Retrievers (10; 7.3%), and Great Danes (7; 5.1%). The median (range) weight was 31.5 kg (6–99.7). The median (range) age was 10.1 years (1.6–15.1).

Malignancy was diagnosed in 15 of 138 (10.9%; 95% CI: 6.2%−17.3%) dogs. Hemangiosarcoma was noted in 11 of 138 (8.0%; 95% CI: 4.0%−13.8%) and were 11 of the 15 (73.3%; 95% CI: 44.9%−99.2%) of the identified malignancies. Splenic stromal cell sarcoma accounted for the remaining 4 (26.7%) spleens with malignancy. Non-malignant changes including nodular hyperplasia (44.9%; 62/138), splenic congestion (29.7%; 41/138), and splenic hematoma (8.0%; 11/138) were also diagnosed.

Lesions were visible on the spleen in most dogs (95/138; 68.8%), as was color change (68/138; 49.3%). No lesions were observed in 33.3% (46/138) of these dogs, and a single lesion was noted in 49.3% (68/138). Two or more lesions were noted in 17.4% of dogs (24/138). The spleen had ruptured in 18 (13.0%) dogs, and splenic rupture was more common in spleens diagnosed with malignancy (5/15; 33.3%; 95% CI: 11.8%−61.6%) than in spleens without a malignancy diagnosis (13/123); 10.6%; 95% CI 5.7%−17.4%) (*P* = 0.013).

In univariate analysis, the prevalence of splenic malignancy did not differ significantly across age or weight quartiles (*P* = 0.171 and *P* = 0.646, respectfully). Malignancy rates significantly differed by the number of visible lesions in the spleen (*P* = 0.003). There were no (0%; 95% CI: 0.0%−7.7%) malignancies in 46 dogs without visible splenic lesions, 13 (19.1%; 95% CI: 10.6%−30.5%) malignancies in 68 dogs with solitary/single visible lesions, 2 (14.3%; 95% CI: 1.8%−42.8%) malignancies in 14 dogs with two visible lesions, and 0 (0%; 95% CI: 0.0%−30.8%) malignancies in 10 dogs with 3 or more visible splenic lesions. Three of the 4 stromal cell sarcomas were in spleens with a single gross lesion, and the other stromal cell sarcoma was in a spleen with two gross lesions. In a multivariable logistic regression analysis assessing age, weight, number of splenic lesions, presence of splenic rupture, and presence of color changes, only the occurrence of splenic rupture was associated with increased odds of malignancy (OR 4.07; 95% CI: 1.06–15.66; *P* = 0.041; Table 1). The multivariable model did not violate the assumptions of the goodness-of-fit.

**Table 1:** Multivariable logistic regression model assessing odds of a histopathologic malignancy diagnosis in splenic tissue submitted post-splenectomy concurrent to GDV correction in 138 dogs.

**Variable**	**Odds ratio (OR)**	**95% confidence interval (CI)**	***P-value***
Splenic rupture – Y/N	4.07	1.06–15.66	0.041^*^
Age – quartile	1.52	0.79–2.90	0.210
Weight – quartile	1.61	0.88–2.95	0.125
Number of visible lesions	1.26	0.58–2.71	0.558
Color change in spleen – Y/N	0.62	0.16–2.44	0.492

^*^Denotes significance.

## Discussion

In this study population, histopathologic malignancy was most associated with spleens that had a solitary discrete mass, with two splenic masses also being associated with malignancy to a lesser degree. However, spleens with three or more masses had no association with a histopathologic diagnosis of malignancy. These findings align with previous studies that have reported that solitary splenic masses are more frequently malignant in cases of primary splenic disease, whereas multifocal splenic changes are more commonly associated with benign conditions such as splenic nodular hyperplasia or splenic hematomas (3–6). Since one or two masses were associated with malignancy but three or more were not, we can only partially accept our first hypothesis.

When performing GDV correction procedures, surgeons may elect to surgically remove the spleen due to concerns regarding splenic devitalization or an overwise abnormal appearance (7). This is likely reflected in the large proportion of dogs initially identified for this study as having received a combined GDV correction and splenectomy without submission of the spleen for histopathologic evaluation. While this resulted in a potential skewing of data for this study due to only 13.5% of dogs meeting all inclusion criteria, it may also emphasize the importance of this study's findings, since the surgeons for those cases may have submitted the tissue if they knew which risk factors to look for. Regardless, since our study found no association between splenic color and malignancy, we cannot reject our second hypothesis.

This result of this study was consistent with an association between splenic rupture and histopathologic diagnosis of malignancy in the splenic tissue removed concurrent to GDV correction. While this was not an objective of the study's design, this finding may provide clinicians with additional guidance when they are deciding whether to submit the removed splenic tissue for histopathology in these cases.

Within this study population, age and body weight did not differ significantly between dogs with benign and malignant splenic disease, which suggests that these demographic factors alone may have limited utility in guiding decisions regarding histopathologic submission. This finding is consistent with previously reported studies that have demonstrated the challenges of predicting splenic malignancy based solely on gross appearance or signalment (8).

In this study population, hemangiosarcoma was the most common cause of splenic malignancy. In primary splenectomy cases, the hemangiosarcoma likelihood prediction (HeLP) score has been described to provide additional context for assessing malignancy risks in dogs presenting with a spontaneous hemoabdomen (9). The HeLP score incorporates body weight, total plasma protein, platelet count, and thoracic imaging findings to estimate the probability of hemangiosarcoma and has been externally validated. To the authors' knowledge, there is no evidence regarding incorporating the HeLP score for dogs presenting with GDV. However, it's possible that incorporating similar risk approaches alongside intraoperative findings, such as the number of splenic lesions, may improve decision-making regarding histopathologic submission of spleens removed during GDV surgery.

Limitations of the current study were inherent of a retrospective study. Primarily, we were unable to retrospectively know why splenic tissue was submitted in some cases and not in others. The impact of the loss of so much of the total case load on our findings is unclear, though this study likely did not identify the true prevalence of malignancy in the cases treated at the study hospitals during the study window. A prospective study with strict requirements for submission of splenic tissue in all cases would provide a more accurate assessment of this question. Additionally, medical records reviewed were determined by searching invoices with a specific procedure code and may have missed potential cases if the invoice was input differently. Finally, some medical records were incomplete or were unable to be accessed for further examination, which may have factored into the results.

In conclusion, the prevalence of splenic malignancy was uncommon within our study population of dogs undergoing GDV correction and concurrent splenectomy. However, malignancy was associated with 1–2 visible splenic lesions, which may provide evidence-based guidance for clinicians as they weigh the risk and financial costs associated with submitting the spleen for histopathologic evaluation. Prospective studies incorporating standardized intraoperative assessment and multivariable analysis are warranted to further refine the decision-making in this clinical setting.

## Data Availability

The raw data supporting the conclusions of this article will be made available by the authors, without undue reservation.
